# Recent GP consultation before death by suicide in middle-aged males: a national consecutive case series study

**DOI:** 10.3399/BJGP.2022.0589

**Published:** 2023-05-03

**Authors:** Faraz Mughal, Lana Bojanić, Cathryn Rodway, Jane Graney, Saied Ibrahim, Leah Quinlivan, Sarah Steeg, Su-Gwan Tham, Pauline Turnbull, Louis Appleby, Roger T Webb, Nav Kapur

**Affiliations:** School of Medicine, Keele University, Keele; honorary clinical research fellow, Division of Psychology and Mental Health, University of Manchester, Manchester; affiliate, NIHR Greater Manchester Patient Safety Translational Research Centre, School of Health Sciences, University of Manchester, Manchester.; National Confidential Inquiry into Suicide and Safety in Mental Health (NCISH), School of Health Sciences, University of Manchester, Manchester.; National Confidential Inquiry into Suicide and Safety in Mental Health (NCISH), School of Health Sciences, University of Manchester, Manchester.; National Confidential Inquiry into Suicide and Safety in Mental Health (NCISH), School of Health Sciences, University of Manchester, Manchester.; National Confidential Inquiry into Suicide and Safety in Mental Health (NCISH), School of Health Sciences, University of Manchester, Manchester.; NIHR Greater Manchester Patient Safety Translational Research Centre, School of Health Sciences, University of Manchester, Manchester.; School of Health Sciences, University of Manchester, Manchester.; National Confidential Inquiry into Suicide and Safety in Mental Health (NCISH), School of Health Sciences, University of Manchester, Manchester.; National Confidential Inquiry into Suicide and Safety in Mental Health (NCISH), School of Health Sciences, University of Manchester, Manchester.; National Confidential Inquiry into Suicide and Safety in Mental Health (NCISH), School of Health Sciences, University of Manchester, Manchester.; NIHR Greater Manchester Patient Safety Translational Research Centre, School of Health Sciences, University of Manchester, Manchester.; NCISH, School of Health Sciences, University of Manchester, Manchester; Manchester Academic Health Sciences Centre, Manchester; Greater Manchester Mental Health NHS Foundation Trust, Manchester; NIHR Greater Manchester Patient Safety Translational Research Centre, School of Health Sciences, University of Manchester, Manchester.

**Keywords:** family practice, general practice, general practitioners, men, primary health care, suicide

## Abstract

**Background:**

Reducing suicide risk in middle-aged males (40–54 years) is a national priority. People have often presented to their GP within 3 months before suicide thus highlighting an opportunity for early intervention.

**Aim:**

To describe the sociodemographic characteristics and identify antecedents in middle-aged males who recently consulted a GP before dying by suicide.

**Design and setting:**

This study was a descriptive examination of suicide in a national consecutive sample of middle-aged males in 2017 in England, Scotland, and Wales.

**Method:**

General population mortality data were obtained from the Office for National Statistics and National Records of Scotland. Information was collected about antecedents considered relevant to suicide from data sources. Logistic regression examined associations with final recent GP consultation. Males with lived experience were consulted during the study.

**Results:**

In 2017, a quarter (*n* = 1516) of all suicide deaths were in middle-aged males. Data were attained on 242 males: 43% had their last GP consultation within 3 months of suicide; and a third of these males were unemployed and nearly half were living alone. Males who saw a GP recently before suicide were more likely to have had recent self-harm and work-related problems than males who had not. Having a current major physical illness, recent self-harm, presenting with a mental health problem, and recent work-related issues were associated with having a last GP consultation close to suicide.

**Conclusion:**

Clinical factors were identified that GPs should be alert to when assessing middle-aged males. Personalised holistic management may have a role in preventing suicide in these individuals.

## INTRODUCTION

In the UK, males aged 40–54 years have the highest age-specific suicide rate.^1^ There are complex interrelated factors that are associated with suicide in these males such as economic pressures, relationship difficulties, and social isolation.^2^^–^4 Reducing suicide risk in middle-aged males is a national policy priority.^5^ Although reducing suicide risk in people who contact mental health services is a key preventive focus, many people do not access these services, and so primary care has an important role in supporting people at risk of suicide.^6^

One study found that among people who died by suicide, 41% had contact with primary care services within 3 months before they died; and 16%, 1 week beforehand.^7^ Between 2003 and 2005 approximately 78% of final GP consultations occurred within 3 months before suicide; and GPs describe reviewing and altering mental health medication during this period.^8^^,^^9^ GPs can identify and support middle-aged males who present in acute distress and/or suicidal crisis, especially in the 3-month window before suicide.^10^^,^^11^ Research is lacking about the characteristics and antecedents of males who died by suicide and had ‘recent contact’ (within 3 months) with a GP before suicide.^12^^,^^13^

The aim of this study was to describe the sociodemographic characteristics and behavioural and clinical antecedents in middle-aged males whose final GP consultation was within 3 months before suicide. Medications prescribed between males with recent GP contact and those who had a GP consultation >3 months before suicide were compared. Antecedents potentially associated with final recent GP contact were explored. By understanding clinical characteristics of males in the recent contact group, potential targets for GP intervention can be identified.

**Table:** How this fits in

Preventing suicide in middle-aged males is a global priority. This national case series study found that 43% of middle-aged males who died by suicide had a final GP consultation in the preceding 3 months, and of these males, over half presented with a mental health problem. Males who had recent GP contact before suicide were more likely to have self-harmed in the 3 months before compared with males who had no recent GP contact. Males who had a current physical illness, recent history of self-harm, attended for a mental health problem, and experienced recent work-related problems were more likely to consult with their GP shortly before dying by suicide. GPs and primary care clinicians should be alert to these clinical factors that may be proximal to suicide, and in turn, offer personalised holistic care.

## METHOD

### Study population

In this national case series study, detailed information was collected about males aged 40–54 years who died by suicide (including probable suicide) between 1 January 2017 and 31 December 2017 in England, Scotland, and Wales. ‘Middle-aged’ was defined as ages 40 to 54 years.

### General population mortality data

The current study used general population mortality data on males who died by suicide for deaths registered in England and Wales from the Office for National Statistics (ONS) and for deaths registered in Scotland, from the National Records of Scotland (NRS). As is standard for suicide research, deaths receiving a conclusion of suicide or intentional self-harm or events of undetermined intent (open verdict) by an HM coroner (England and Wales) or by a procurator fiscal (Scotland) when a death is registered were included. Deaths with International Classification of Diseases, Tenth Revision (ICD-10) codes X60– X84, Y10–Y34 (excluding Y33.9), and Y87 were included.^14^ Deaths that were summarised by narrative conclusion at coronial inquest were included if ONS procedures applied one of the ICD-10 codes listed above in England and Wales only.

### Sample

In 2017, 1516 males aged 40–54 years died by suicide in England, Scotland, and Wales. The authors aimed to obtain information on the antecedents of suicide in these deaths from official investigations on a stratified, random sample of 20% of these males. A fifth of the cases of all individuals were sampled to ensure that sufficient data were obtained and that the research team was adequately resourced to conduct the investigation. This 20% sample was stratified according to the proportion of deaths in each of these groups:

- by 5-year age band (40–44, 45–49, and 50–54 years) within the broader 40–54 years range; and
- by country (England, Wales, and Scotland).

Using the SPSS random allocator function, the authors selected the sample from the total number of deaths by suicide that had been notified by national data providers at the time of sampling (*n* = 1486). In total, 288 (19%) middle-aged males were sampled. Information was obtained about the circumstances surrounding their death, the stressors close to suicide, and their contact with primary care from the data sources that are described below.

### Data sources

#### Coronial inquest hearings/files or police death reports

Audiorecordings of coronial inquest proceedings were requested from senior coroners in the jurisdictions of all sampled deaths in England and Wales. If unavailable, inquest depositions or statements were requested. Redacted police death reports were requested from the Crown Office and Procurator Fiscal Service for all sampled deaths that occurred in Scotland. Information was attained for 228 (79%) of the sampled 288 suicide deaths: in 12 deaths the coroner or equivalent was unable (*n* = 5) or did not wish to provide the data (*n* = 7); for 48 deaths, data were not returned on time.

#### National Confidential Inquiry into Suicide and Safety in Mental Health (NCISH) data

The NCISH collects data on a complete UK-wide consecutive case series of people who died by suicide within 1 year of being seen by mental health services. Detailed NCISH data- collection methods have been published.^15^ NCISH data were obtained for 86/288 (30%) of the sampled deaths.

#### NHS serious incident reports

Where a patient suicide was identified from NCISH data, a copy of the serious incident report about factors that led to the suicide was requested from the medical director of the treating NHS trust/health board. Reports from 68 deaths (24%) were obtained. The deaths of 12 males did not meet organisational review criteria, and data about six deaths were not returned on time.

#### Criminal justice system reports

In England and Wales, the Prison and Probations Ombudsman release independent fatal incident investigation reports of deaths by apparent suicide in custody. The Prison and Probations Ombudsman website was searched for reports for males who died in custody. In Scotland, certain types of death are investigated at fatal accident inquiries (FAI), including deaths in prisons. The judgements of FAIs are published on the Scottish Courts and Tribunals website. The Scottish Courts and Tribunals website was searched to locate FAIs relating to suicide in males who died in custody. Three reports of suicides were identified across England, Wales, and Scotland.

### Procedures

Information about antecedents of suicide were extracted using a pre-defined proforma informed by literature and patient and public involvement (PPI), and then transferred into a standardised database for aggregate analysis. Information was collected on demographic factors (relationship status, employment status, and living circumstances), medical history (physical health conditions, alcohol misuse, and illicit drug use), psychiatric history (psychiatric disorders and medication), disclosure of suicidal ideas and/or intent, history of self-harm, and recent events (problems with family, work, finance, or accommodation in the 3 months before death). The last known contact with GPs and secondary care (emergency department and mental health) was recorded.

The authors recorded factors when they were referred to in any data source as having been present in the individual’s life at any time, or specifically in the 3 months before death (definition of ‘recent’). Reference to an antecedent factor (see Supplementary Table S1 for definitions) at an investigation suggests that it was seen as relevant to the death.

To ensure interrater reliability of data extraction, approximately a tenth of cases (*n* = 30) were reviewed by three of the authors and a Fleiss’ Kappa reliability test performed.^16^ Initial levels of agreement were 58% to 100%. Upon disagreement, information was independently re-evaluated and discussed until agreement reached: concordance increased to 100%.

### PPI

Three male members of the Mutual Support for Mental Health Research PPI group with lived experience of suicidal distress informed the development of the data extraction proforma and contributed to interpretation of findings.

### Statistical analysis

If an antecedent was not mentioned in a data source, the authors assumed that it was unlikely to have been present and it was recorded as absent/not relevant. Pearson’s χ^2^ or Fisher’s exact test were used to test for associations between subgroups. Antecedents of males in the recent final GP consultation group before suicide were examined using descriptive analysis and compared with males who did not have recent GP contact before suicide, including no GP contact at all.

A subgroup analysis comparing medications prescribed in males with a recent GP contact versus males whose GP consult was >3 months before death was conducted. Univariate logistic regression models were initially fitted with final recent GP consultation before death by suicide as the outcome variable. A multivariable model was then generated using a backwards elimination variable selection approach: the variable with the highest *P*-value was deleted first.^17^ Results with *P*<0.05 (two-sided) were considered statistically significant. Analyses were undertaken in Stata (version 16.1). When reporting results, cell counts below three (including zero) were supressed, in accordance with ONS guidance on disclosure control to protect confidentiality.^18^ Results from England, Wales, and Scotland are presented as aggregate values.

## RESULTS

The NCISH was notified through ONS and NRS of 5950 deaths by suicide in England, Wales, and Scotland that occurred between 1 January 2017 and 31 December 2017, with 1516 deaths among males aged 40–54 years. This demographic subgroup constituted a quarter (*n* = 1516/5950, 25%) of all deaths by suicide, and more than one- third (*n* = 1516/4458, 34%) of all suicides among males. The most common method of suicide was by hanging or strangulation (*n* = 932, 61%) followed by self-poisoning (*n* = 227, 15%), with a fifth of suicide deaths by self-poisoning being from opioid/opiate substances (*n* = 45, 20%). Information about the sociodemographic characteristics and antecedents of suicide deaths was obtained for 242 (84%) of the 288 middle-aged males in this study’s sample. The mean age of these males was 47 (40–54) years, and they were from England (*n* = 193), Scotland (*n* = 34), and Wales (*n* = 15). Information was attained mostly from coronial inquest hearings or police death reports (*n* = 228).

In total, 90% (*n* = 219/242) of males in the sample were registered with a general practice when they died by suicide. Overall, 12% (*n* = 29) of the 242 males had a final consultation with a GP during the preceding week before death; 31% (*n* = 76) between day 8 to 12 weeks, and 27% (*n* = 66) >3 months before. In 12% of males (*n* = 28), the time since last GP consultation before suicide was unspecified, and in 18% (*n* = 43) last GP contact was not indicated. From males with a known time of final GP consultation (*n* = 171/242) before suicide, 61% (*n* = 105) last consulted a GP within 3 months (including 1 week), and 39% (*n* = 66) >3 months before suicide (Table 1).

**Table 1.** Subgroup analysis of prescribed medications in males and the relationship with time of last GP consultation before suicide (*n* = 171)

**Prescribed medication**	**Last GP consultation before suicide, *n* (%)**		**χ^2^**	***P*-value**
	**GP consultation within 3 months (*n* = 105)**	**GP consultation >3 months before (*n* = 66)**		
**SSRI/SNRI and related**	56 (53)	15 (23)	15.634	<0.001^a^
**Oral antipsychotic**	20 (19)	5 (8)	4.272	0.039^b^
**Depot antipsychotic**	<3 (2)	4 (6)	—	0.156^c^
**Tricyclic antidepressant**	7 (7)	3 (5)	—	0.414^c^
**Lithium/mood stabilisers**	4 (4)	3 (5)	—	0.551^c^
**Other antidepressants**	14 (13)	8 (12)	0.053	0.818
**Benzodiazepines**	15 (14)	3 (5)	—	0.035^b,c^
**Other psychotropic drugs**	27 (26)	3 (5)	—	<0.001^a,c^
**Opiate for pain relief**	14 (13)	4 (6)	—	0.103^c^

a *P*<*0.01.*

b *P*<*0.05.*

c *One-sided Fisher’s exact test. SNRI = serotonin–noradrenaline reuptake inhibitor. SSRI = selective serotonin reuptake inhibitor.*

### Final recent GP contact compared with no recent GP contact before suicide

In total, 43% (*n* = 105/242) of males had a last recent GP consultation before dying by suicide. As shown in Table 2, one-third of these males were unemployed at that time. Sixty-three percent were single, divorced, separated, or widowed. Nearly half were living alone. Of males who recently saw a GP before dying by suicide, 8% were from an ethnic minority background.

**Table 2.** Sociodemographic, behavioural, and clinical characteristics of middle-aged males who died by suicide with a known recent GP consultation before suicide (*n* = 242)

**Characteristic**	**Recent last GP consultation *n* (%) (*n* = 105)**	**Did not have recent last GP consultation, *n* (%) (*n* = 137)**	***P*-value**
**Sociodemographic**			
Single	38 (36)	61 (45)	0.191
Unemployed	35 (33)	37 (27)	0.286
Living alone	49 (47)	60 (44)	0.656
Ethnic minority group	8 (8)	12 (9)	0.750

**Behavioural**			
History of self-harm within 3 months of death	33 (31)	13 (9)	<0.001^a^
History of self-harm >3 months before death	23 (22)	38 (28)	
History of suicidal thoughts	34 (32)	28 (20)	0.035^b^
History of alcohol misuse	37 (35)	51 (37)	0.750
History of drug misuse	33 (31)	41 (30)	0.802

**Clinical**			
Major physical illness at time of death	54 (51)	46 (34)	0.005^a^
Respiratory disease	12 (11)	15 (11)	0.906
Chronic pain	13 (12)	9 (7)	0.119
Musculoskeletal disease	6 (6)	4 (3)	0.224^c^

**Psychiatric diagnosis**			
Depressive or anxiety illness	42 (40)	39 (28)	0.060
Schizophrenia/delusional disorders	10 (10)	9 (7)	0.397
Alcohol or drug misuse	13 (12)	13 (9)	0.472

**Reasons for last GP contact**			
Mental health or psychological problem	54 (51)	21 (15)	<0.001^a^
Alcohol and/or drug misuse	7 (7)	7 (5)	0.607

**Outcome at last GP consult**			
GP consultation only	35 (33)	29 (21)	0.033^b^
Referral	22 (21)	10 (7)	0.002^a^

**Last emergency department attendance within 3 months**	23 (22)	12 (9)	0.004^a^

**Social events in the 3 months before suicide**			
Separation from partner	24 (23)	24 (18)	0.302
Social isolation	14 (13)	10 (7)	0.120
Recent work-related problems (including on sick leave)	30 (29)	15 (11)	<0.001^a^
In debt	17 (16)	18 (13)	0.504

a *P*<*0.01.*

b *P*<*0.05.*

c *One-sided Fisher’s exact test.*

Males whose GP consultation before suicide was recent were more likely than males who had no recent GP contact to have had a history of self-harm within 3 months before suicide and to have had a history of suicidal ideation. They were also significantly more likely to have had a major physical illness (Table 2).

Males who had a recent GP consultation before suicide were more likely than males who had not to have presented to a GP with a mental health or psychological problem. From the outcomes of last GP consultation (either patient’s mental health team informed, GP consultation only, referral to other services, other specified reason, or not applicable), males who had a recent consultation were more likely to have had a GP consultation with no other service involvement and they were more likely to have been referred in their last GP consultation. Males who had a recent GP consultation before suicide were more likely to report experiencing recent work- related problems, including being on sick leave. Males with a recent GP consultation were also more likely to have presented to a hospital emergency department during the 3 months before suicide (Table 2).

### Prescribed medications in recent compared with >3-month GP contact group

As listed in Table 1, males who saw a GP recently were more likely to have been prescribed a selective serotonin reuptake inhibitor (SSRI)/serotonin– noradrenaline reuptake inhibitor (SNRI) type antidepressant, oral antipsychotic, benzodiazepine, or other psychotropic medication by a GP, mental health, or emergency department clinician (see Supplementary Table S2 for medication list), compared with males who saw a GP >3 months before suicide.

### Clinical predictors of final recent GP contact

All significant variables in Table 2 (except GP consultation outcome variables) were entered into model A simultaneously and then insignificant variables were removed individually until the final model was fitted. In model D (*n* = 242), having a major physical illness, recent self-harm, presenting for a mental health or psychological problem, and recent problems in the workplace (including being on sick leave, bullying, or a change or loss of job) was associated with males who had a GP consultation within 3 months of suicide (Table 3). The fit of models (likelihood ratio test) improved from 66.67 (model A) to 63.65 (model D).

**Table 3.** Results of backwards elimination logistic regression for middle-aged males who died by suicide with final GP consultation before suicide (*n* = 242)

**Characteristic**	**Model A**			**Model B**			**Model C**			**Model D**		
	**OR**	**95% CI**	***P*-value**	**OR**	**95% CI**	***P*-value**	**OR**	**95% CI**	***P*-value**	**OR**	**95% CI**	***P*-value**
**Constant**	0.22	0.13 to 0.38	<0.001^a^	0.23	0.14 to 0.38	<0.001^a^	0.23	0.14 to 0.38	<0.001^a^	0.21	0.13 to 0.34	<0.001^a^
**History of self-harm within 3 months**	2.53	0.98 to 6.50	0.054	2.53	0.98 to 6.49	0.054	2.89	1.27 to 6.56	0.011^b^	3.49	1.60 to 7.61	0.002^a^
**History of self-harm >3 months ago**	0.55	0.26 to 1.16	0.116	0.54	0.26 to 1.15	0.11	0.55	0.26 to 1.16	0.117	—	—	—
**History of suicidal thoughts**	1.15	0.57 to 2.33	0.69	—	—	—	—	—	—	—	—	—
**Major physical illness**	2.62	1.42 to 4.83	0.002^a^	2.62	1.42 to 4.84	0.002^a^	2.63	1.43 to 4.84	0.002^a^	2.39	1.32 to 4.32	0.004^a^
**Last GP consult for mental health/psychological problem**	5.50	2.70 to 11.20	<0.001^a^	5.74	2.90 to 11.36	<0.001^a^	5.66	2.87 to 11.15	<0.001^a^	4.92	2.58 to 9.37	<0.001^a^
**Recent work-related problems (including on sick leave)**	2.99	1.37 to 6.54	0.006^a^	2.98	1.37 to 6.51	0.006^a^	3.09	1.43 to 6.67	0.004^a^	3.07	1.43 to 6.61	0.004^a^
**Recent emergency department attendance within 3 months**	1.34	0.48 to 3.75	0.578	1.34	0.48 to 3.74	0.576	—	—	—	—	—	—

a *P*<*0.01.*

b *P*<*0.05.*

*OR = odds ratio. As seen across Models A to D the variable with the highest* P*-value was deleted first (backwards elimination variable selection approach) till the final Model.*

## DISCUSSION

### Summary

This study found that 43% of males saw a GP within 3 months of suicide and of these males, over half presented to the GP with a mental health or psychological problem. Males who last saw a GP in the 3 months before suicide were more likely than those who had not to have a recent history of self-harm. Males who saw a GP within 3 months of death were more likely to have been taking an SSRI/SNRI antidepressant, oral antipsychotic, benzodiazepine, or other psychotropic medication compared with males who saw a GP >3 months before suicide. Males who had a major physical illness, recent self-harm, presented for a mental health problem, and had work-related issues were more likely to have had recent GP contact before suicide.

### Strengths and limitations

This is the first study, to the authors’ knowledge, to have examined antecedents of suicide in middle-aged males who had recent GP consultations before suicide. Data predominantly came from coroners who independently obtain evidence from several sources including personal narratives of families, friends, and professionals in contact with males before suicide. Males with lived experience were involved in the study, improving the credibility of the findings.

There are, however, several limitations. This was a case series study thus causal inferences cannot be made about observed relationships. Males who had a major physical illness or work-related problems may have been frequently seeing a GP and therefore more likely to have had a recent GP consult close to suicide. The findings are aggregated for England, Wales, and Scotland; and in turn will be driven by a larger number of suicide deaths in England.

Ethnicity was poorly recorded therefore likely underreported. The authors may have underestimated the true figure for some antecedents, particularly if they were viewed as sensitive (for example, separation from partner); and other figures may be overestimates as families/friends search for meaning following deaths and may focus on self-perceived relevant factors.^19^ Some data may have been influenced by recall bias. Data were not specifically collected on the number or frequency of GP consultations and so it was not possible to identify patterns in GP consultations before suicide. This study does not tell us about final recent GP consultations during COVID-19.

### Comparison with existing literature

Stanistreet *et al* compared health service contacts before suicide and accidental deaths in young males (aged 15–39 years) in 1995 and found that 56% (*n* = 45/80) of males who died by suicide had seen a GP in the 3 months before suicide;^20^ the current study found 43% of males had their final GP consultation within 3 months before suicide. A Norwegian study of suicide deaths from 2006 to 2015 found that 33% of males aged 30–44 years and 39% of males aged 45–59 years had a GP consultation 1 month before suicide highlighting an opportunity for GP intervention in the month before suicide.^13^ Among French males who visited their family doctor during 2019, 24% had a work-related mental health problem;^21^ the current study found males who had recent GP contact before suicide were more likely to have experienced recent work-related stress compared with those who had not, highlighting the importance of enquiring about work-related problems. Risk factors identified in general practice for suicide in males include past self-harm and major life stresses: the current study found males who had a recent GP consult were more likely to have recently self-harmed.^22^

### Implications for research and practice

The current findings contribute new evidence that GPs should consider when managing middle-aged males. This study identified antecedents such as current major physical health illness and a recent history of self- harm that are more likely to occur in males who consult a GP 3 months before suicide than those who had not. It is important to examine the associations of antecedents before suicide and the timing of GP contact in the 3-month window. Exploring the mental health impact of having a major physical illness or experiencing work-related problems can lead to understanding about how GPs can intervene. Acceptable ways GPs can acutely intervene to reduce the chances of suicide in middle-aged males need to be researched.

Suicide in a middle-aged male may be a rare occurrence for GPs; but a patient suicide can have a detrimental effect on a GP’s wellbeing.^23^ Males in midlife have the highest suicide rates in the UK, and with COVID-19 exacerbating known suicide risks it is crucial GPs are alert to identified antecedents that are more likely in males who died by suicide with recent GP contact. Males who present with an identified antecedent such as a mental health problem, recent self-harm, or suicidal ideation should receive a risk formulation, focusing on clinical needs and tailoring treatment to needs using a strengths-based approach. The 2022 National Institute for Health and Care Excellence self-harm guidance states that risk stratification into low, medium, or high risk to predict future suicide or repetition of self- harm should not be used.^24^

GPs attempting to implement the ‘Making Every Contact Count’ approach^25^ (delivering healthy lifestyle messages to encourage behaviour change, for example, about alcohol intake or stress-reduction techniques, and directing to appropriate services) could potentially prevent suicide in midlife males, specifically for males with a major physical illness, recent self-harm, work-related problems, and who present with a mental health problem.^6^ Longer-term intervention can include referral to mental health services, talking therapies, or third-sector teams; self- help resources; and treatment of underlying mental illness.^10^

Males prescribed an SSRI/SNRI antidepressant, oral antipsychotic, or benzodiazepine were more likely to have seen their GP within 3 months of suicide, which may indicate that mental health reviews were conducted or there was a deterioration in their mental health. In practice it is important to recognise this and explore self-harm and suicidal thoughts in males taking these medications.

GPs may consider allocating more time or arranging follow-up appointments to carefully assess middle-aged males, in particular for those who present with a new mental health problem or work-related problems. Preventing suicide in middle-aged males remains a national priority and GPs have a key role, especially in early assessment and intervention, in a system-wide approach to suicide prevention in these individuals.^26^

## Data

Data from this study cannot be shared because of information governance restrictions in place to protect confidentiality. A request to access data can be made to the HQIP: https://www.hqip.org.uk/national-programmes/accessing-ncapop-data/#.ZEAOsOzMKAM
